# OA21 The Rising Ferritin – Recognising HLH Secondary to Still’s Disease in Transitional Rheumatology Care

**DOI:** 10.1093/rap/rkag113.021

**Published:** 2026-11-03

**Authors:** Hugh Larkin, Eleana Ntatsaki, Suzanne Lane, Diomidis Ioannidis

**Affiliations:** East Suffolk and North Essex NHS Foundation Trust, Ipswich, United Kingdom; East Suffolk and North Essex NHS Foundation Trust, Ipswich, United Kingdom; East Suffolk and North Essex NHS Foundation Trust, Ipswich, United Kingdom; East Suffolk and North Essex NHS Foundation Trust, Ipswich, United Kingdom

## Abstract

**Introduction:**

Haemophagocytic lymphohistiocytosis (HLH) is a rare hyperinflammatory syndrome with high mortality if not recognised and treated promptly. Adult-onset Still’s disease (AOSD) and systemic juvenile idiopathic arthritis (sJIA) are recognised causes of secondary HLH in adults and children, respectively. Overlapping clinical features with infection and malignancy can delay diagnosis and worsen outcomes.

We present the case of a 17-year-old admitted to an adult medical ward with a febrile illness, highlighting the diagnostic process and management. This case explores the challenges of transitional care and whether Still’s disease presents differently in teenagers than in older adults.

**Case description:**

A 17-year-old female attended hospital with a four-week history of rash, high fever and joint pain.

Clinical examination was notable for an extensive macular rash, tenderness over the wrists and knees without overt synovitis, and palpable cervical adenopathy.

Bloods revealed a normocytic anaemia and neutrophil-predominant leukocytosis. Serological testing for connective tissue disease, vasculitis and inflammatory arthritis was negative. Ferritin at admission was <1,000 ug/L. Extensive investigation during the first week of admission did not reveal a source of infection or solid organ malignancy. A diagnosis of Adult Onset Still’s Disease was made and moderate-dose oral corticosteroid was initiated. Despite this the patient continued to spike high temperatures daily. By day eight of admission, the ferritin had risen to > 50,000 ug/L. Concern was raised for HLH complicating Still’s disease. Treatment with IV methylprednisolone and anakinra was initiated. Within 24 hours the fever and arthralgia had settled. Within 72 hours she was symptom-free, the CRP had normalised and the ferritin fell to < 10,000.

At 2-, 12- and 24-week follow-up she remained symptom-free and with normal inflammatory markers.

**Discussion:**

HLH is a rare disease but the challenges encountered by clinicians caring for patients with HLH are common. These range from the logistical challenge of obtaining time-critical diagnostic investigations, limited familiarity with HLH among non-specialist clinicians working out-of-hours, and the need for cross-specialty agreement on the key decision to initiate HLH treatment. All the above is exacerbated further by the fact that HLH is notoriously difficult to recognise because its core features overlap with more common disease aetiologies: Sepsis and malignancy in the critically ill patient can lead to inflammation, cytopenias and ferritin elevation.

Our discussion focuses on the practical decision-making that led to a successful outcome. We compare the presentation of Still’s disease across the paediatric- adult spectrum, distinguishing marked hyperferritinaemia associated with Still’s from evolving HLH, and consider an age-appropriate approach to excluding malignancy while limiting radiation exposure in this patient group. We also reflect on the challenges of communicating diagnostic uncertainty and high-risk treatment decisions with a young patient and her family. A graphical timeline of clinical features, laboratory parameters and treatment interventions illustrates how serial assessment was central to establishing this patient’s diagnosis.

**Key learning points:**

• Diagnosing Still’s disease in young adults can be a challenge: Clinical features vary across the age spectrum, while diagnostic criteria for sJIA and AOSD remain traditionally distinct despite representing a disease continuum.

• Serial ferritin measurements interpreted alongside evolving clinical features and the H-score, can facilitate earlier recognition of secondary HLH in patients admitted with suspected Still’s disease.

• Transitional-age patients presenting with rheumatological emergencies benefit from early multidisciplinary collaboration. Clear, age-appropriate communication and patient education are essential to support shared decision-making, safe discharge and successful long-term follow-up.

